# Role of the BaeSR two-component system in the regulation of *Acinetobacter baumannii adeAB* genes and its correlation with tigecycline susceptibility

**DOI:** 10.1186/1471-2180-14-119

**Published:** 2014-05-09

**Authors:** Ming-Feng Lin, Yun-You Lin, Hui-Wen Yeh, Chung-Yu Lan

**Affiliations:** 1 Department of Medicine, National Taiwan University Hospital Chu-Tung Branch, Hsin-Chu County, Taiwan; 2 Institute of Molecular and Cellular Biology, National Tsing Hua University, Hsin-Chu City, Taiwan; 3 Laboratory Medicine Division, National Taiwan University Hospital Hsin-Chu Branch, Hsin-Chu City, Taiwan; 4 Department of Life Science, National Tsing Hua University, Hsin-Chu City, Taiwan

**Keywords:** *Acinetobacter baumannii*, Tigecycline, Two-component regulatory system, Efflux pumps

## Abstract

**Background:**

Tigecycline resistance in *Acinetobacter baumannii* is primarily acquired through overexpression of the AdeABC efflux pump. Besides AdeRS, other two-component regulatory systems (TCSs) involving the regulation of this transporter have not been clarified.

**Results:**

In this study, we found that the TCS genes *baeR* and *baeS* are co-transcribed and function as stress responders under high osmotic conditions. The *baeSR* and *adeAB* genes showed increased transcription in both the laboratory-induced and clinical tigecycline-resistant strains compared with the wild-type strain. The deletion of *baeR* in the ATCC 17978 strain led to 67–73% and 68% reduction in *adeA* and *adeB* expression, respectively, with a resultant 2-fold decrease in the tigecycline minimal inhibition concentration (MIC). In contrast, the overexpression of *baeR* resulted in a doubled tigecycline MIC, with a more than 2-fold increase in *adeA* and *adeB* expression. The influence of *baeR* knockout on *adeAB* gene expression can also be observed in the laboratory-induced tigecycline-resistant strain. A time-kill assay showed that the *baeR* deletion mutant showed an approximate 1-log_10_ reduction in colony forming units (CFUs) relative to the wild-type strain when the tigecycline concentration was 0.25 μg/mL throughout the assay period. The wild-type phenotype could be restored by trans-complementation with pWH1266-*kan*^*r*^-*baeR*. Increasing the tigecycline concentration to 0.5 μg/mL produced an even more marked 4.7-log_10_ reduction in CFUs of the *baeR* deletion mutant at 8 h, while only a 2.1-log_10_ reduction was observed for the wild-type strain.

**Conclusions:**

Taken together, these data show for the first time that the BaeSR TCS influences the tigecycline susceptibility of *A. baumannii* through the positive regulation of the resistance-nodulation-division efflux pump genes *adeA* and *adeB*.

## Background

*Acinetobacter baumannii* has emerged as a major cause of nosocomial infections, especially in intensive care units [1]. Both its ability to acquire resistant determinants and to adapt to harsh environments has made *A. baumannii* a successful pathogen [2]. *A. baumannii* has high rates of resistance to many available antibiotics in clinical practice. For example, imipenem-resistant *A. baumannii* constituted > 50% of a worldwide collection of clinical samples between 2005 and 2009 [3]. A Taiwanese surveillance report of antimicrobial resistance in 2000 found that 73% of *A. baumannii* isolates collected from 21 medical centers and regional hospitals were ceftazidime-resistant [4]. Therefore, there are only a few effective anti-*Acinetobacter* drugs currently available, including polymyxins and tigecycline [5]. Tigecycline is the first drug from the glycylcycline class, a new class of antibiotics derived from tetracycline [6]. Tigecycline acts as a protein synthesis inhibitor by binding to the 30S ribosomal subunit, and thus blocking entry of the tRNA into the A site of the ribosome during translation. Although tigecycline has an expanded spectrum of antibacterial activity, previous studies have shown that tigecycline resistance has emerged in *A. baumannii*. Resistance in these strains is associated with multidrug efflux systems, especially the overexpression of the *adeABC* genes, which encode an efflux pump [7,8]. The AdeABC pump belongs to the resistance-nodulation-division (RND) family, which has a three-component structure [9].

Bacterial two-component systems (TCSs) play an important role in the regulation of adaptation to and signal transduction of environmental stimuli, including stress conditions [10]. TCSs are typically composed of a membrane-localized sensor with histidine kinase activity and a cytoplasmic response regulator (RR). Generally, upon sensing environmental changes, signaling begins via autophosphorylation of the sensor protein at a conserved histidine residue. The phosphate is then transferred to an aspartic acid residue in the so-called receiver domain of the corresponding RR. Phosphorylation may induce conformational changes in RRs, which alters their DNA- binding properties, thus modulating downstream gene expression [11]. Importantly, the roles of TCSs in the regulation of antimicrobial resistance have recently been documented in several species of bacteria [12-14]. Additionally, the AdeS-AdeR TCS controls genes encoding the AdeABC pump in *A. baumannii*[15]. AdeS is a sensor kinase, whereas AdeR is an RR. Point mutations in AdeS and AdeR, or a truncation of AdeS due to an IS*Aba1* insertion, may be related to the overexpression of AdeABC, which leads to multidrug resistance [15,16]. However, the existence of *adeABC*-overexpressing mutants without any mutations in *adeRS*[7] and the low expression of *adeABC* in a clinical strain of *A. baumannii* with the IS*AbaI* insertion in the *adeRS* operon [16] suggest that the regulation of *adeABC* gene expression is complicated, and other regulatory mechanisms may be involved.

BaeSR is a TCS and is one of the five extracytoplasmic response pathways in *Escherichia coli*. BaeSR detects environmental signals and responds by altering the bacterial envelope [17]. The main function of the Bae response is to upregulate efflux pump expression in response to specific envelope-damaging agents [18]. Indole, flavonoids, and sodium tungstate have been shown to be novel inducers of the BaeSR response [18,19]. In *Salmonella typhimurium*, one of the physiological roles of BaeR is to respond to stresses that specifically damage MdtA, leading to an induction of MdtA transport and the removal of the toxic agent (e.g., tungstate waste) from the cell [19]. In *TolC* mutants or efflux mutants of *E. coli*, the overexpression of *spy*, which encodes a periplasmic chaperone, depends on the BaeRS and CpxARP stress response systems [20]. A genome-wide analysis of *E. coli* gene expression showed that BaeR overproduction activates genes involved in multidrug transport, flagellum biosynthesis, chemotaxis, and maltose transport [21]. Furthermore, BaeSR is also able to activate the transcription of the *yegMNOB* (*mdtABCD*) transporter gene cluster in *E. coli* and increases its resistance to novobiocin and deoxycholate [22]. Because there is a potential similarity in the biological functions of *mdtABCD* in *E. coli* and *adeABC* in *A. baumannii*, we here explore the role of BaeSR in the regulation of the transporter gene *adeAB* in *A. baumannii* and report the positive regulation of these factors, which leads to increased tigecycline resistance.

## Results

### Sequence analysis of the AdeAB efflux pump and the BaeR/BaeS TCS

A search of the GenBank database (http://www.ncbi.nlm.nih.gov/genbank) revealed that, similar to other strains of *A. baumannii*, the ATCC 17978 strain contains sequences encoding the AdeABC-type RND efflux pump. There are two *adeA* genes (A1S_1751 and A1S_1752) and one *adeB* gene (A1S_1750) in the genome; however, no *adeC* gene was found. AdeB is a transmembrane component with two conserved domains: the hydrophobe/amphiphile efflux-1 (HAE1) family signature and a domain conserved within the protein export membrane protein SecD_SecF. Both AdeA proteins are inner membrane fusion proteins with biotin-lipoyl-like conserved domains. We designated A1S_1751 as AdeA1 and A1S_1752 as AdeA2 for differentiation.

**Figure 1:**
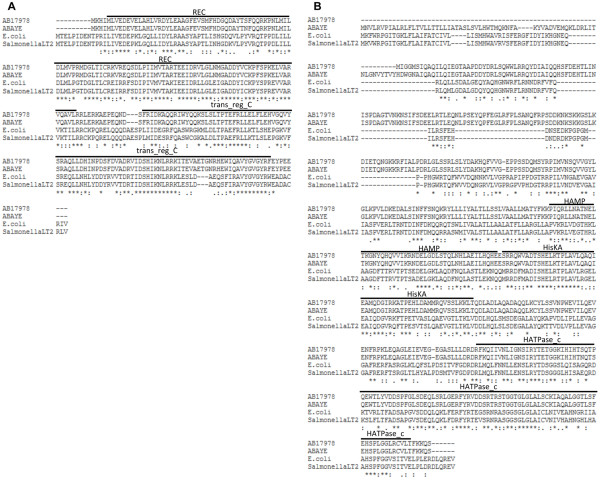
**Sequence alignment of BaeR and BaeS from *Acinetobacter baumannii *ATCC 17978 and other bacteria. (A)** Sequence alignments of *A. baumannii* A1S_2883 with BaeR homologs in other bacteria revealed that A1S_2883 shares 64.6% similarity with BaeR of *Escherichia coli* and 65.2% similarity with BaeR of *Salmonella* LT2. **(B)** A1S_2884 shares 48.1% similarity with BaeS of *E. coli* and 46.3% similarity with BaeS of *Salmonella* LT2. REC and trans_reg_C are two conserved domains of BaeR, while HAMP, HisKA, and HATPase_c are conserved domains of BaeS. AB17978: *A. baumannii* ATCC 17978, ABAYE: *A. baumannii* AYE, E. coli: *E. coli* str. K-12 substr. MG1655, SalmonellaLT2: *Salmonella enterica* subsp. *enterica* serovar *Typhimurium* str. LT2.

The *A. baumannii* ATCC 17978 gene A1S_2883 encoded a protein of 228 amino acids. Sequence alignments of *A. baumannii* A1S_2883 with BaeR homologs in other bacteria showed that A1S_2883 shared 64.6% similarity with BaeR of *E. coli* str. K-12 substr. MG1655 and 65.2% similarity with BaeR of *Salmonella enterica* subsp. *enterica* serovar *Typhimurium* str. LT2 (Figure 1A). In addition, protein analysis using Prosite (http://prosite.expasy.org/) predicted that *A. baumannii* A1S_2883 contained a response regulatory domain at amino acid residues 3 to 115 and a phosphorylation site at amino acid residue 51 (aspartate). Therefore, the role of A1S_2883 may be similar to that of BaeR in other bacterial species; thus, we have designated A1S_2883 as BaeR in *A. baumannii*.

*A. baumannii* ATCC 17978 gene A1S_2884 encodes a protein of 487 amino acids. Sequence alignments showed that A1S_2884 shared 48.1% similarity with BaeS of *E. coli* str. K-12 substr. MG1655 and 46.3% similarity with BaeS of *Salmonella enterica* subsp. *enterica* serovar *Typhimurium* str. LT2 (Figure 1B). Protein analysis using Prosite predicted that *A. baumannii* A1S_2884 contains a HAMP (histidine kinase, adenylyl cyclase, methyl-accepting protein, and phosphatase) and a histidine kinase domain at amino acid residues 214 to 266 and 274 to 487, respectively. In addition, the histidine at residue 277 is predicted to be a phosphorylation site. Therefore, the role of A1S_2884 may be similar to that of BaeS in other bacterial species; thus, A1S_2884 is designated as BaeS in *A. baumannii*.

### Co-transcription of *baeR* and *baeS* as an operon

**Figure 2:**
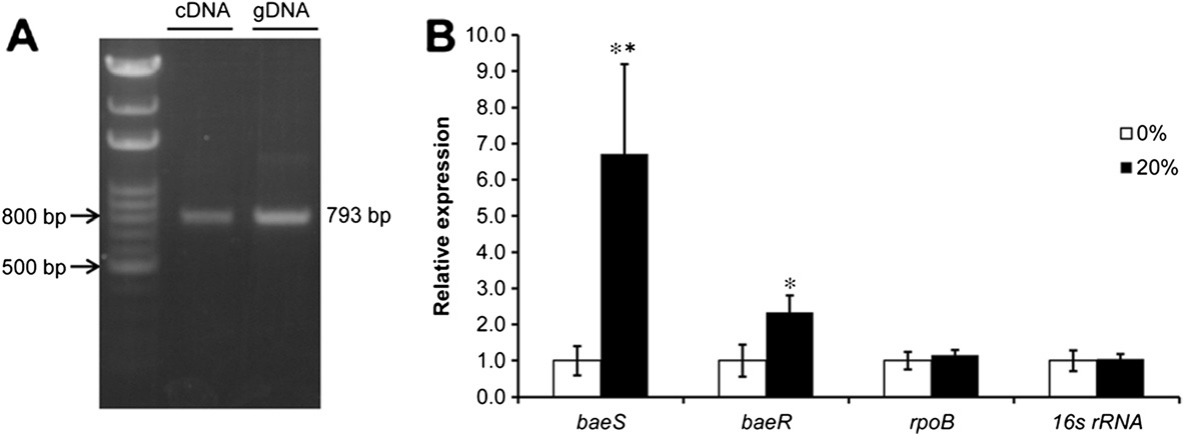
**Evaluation of *baeR *and *baeS *expression. (A)** The co-transcription of *baeR* and *baeS* was determined by agarose gel electrophoresis of the product obtained by reverse transcription polymerase chain reaction (RT-PCR). Lane 2 (cDNA) and 3 (genomic DNA) reveal a 793-bp DNA fragment covering the junction between both the *baeR* and *baeS* genes. **(B)** The relative transcript levels of *baeR* and *baeS*, as determined by RT-PCR, under different osmolarity conditions. The cells were grown on Luria-Bertani (LB) agar with or without 20% sucrose (37°C, 220 rpm). *16S rRNA* and *rpoB* genes were used as controls. The expression levels of *baeR* and *baeS* were 2.3- and 6.7-fold higher in cells experiencing osmotic stress than those in cells grown without sucrose. The results are displayed as the means ± SD from four independent experiments. *, *P* < 0.05; **, *P* < 0.01.

Although the BaeSR TCS has been characterized in *E. coli*[21,22], the biological functions of BaeSR in *A. baumannii* have not been revealed. Genome analysis of *A. baumannii* ATCC 17978 shows that the coding sequences of *baeR* (A1S_2883) and *baeS* (A1S_2884) are arranged sequentially, suggesting that the two genes may be co-transcribed as an operon. To test this hypothesis, reverse transcription-polymerase chain reaction (RT-PCR) was performed using the primers *baeS-*co F and *baeS-*co R (Table 1). As shown in Figure 2A, a 793-bp DNA fragment covering the junction between both *baeR* and *baeS* was amplified by RT-PCR. We concluded that *baeR* and *baeS* are co-transcribed as a single operon in *A. baumannii* ATCC 17978.

**Table 1:** Oligonucleotides used in this study

**Primer name**	**Sequence (5′ to 3′)**^**a**^
*baeS*-co_F	CGCGTAGTACAGGTGGAACA
*baeR*-co_R	TCCACTCATGACGGTTACCA
*kan*^*r*^-*Bam*HI_F	ATAT**GGATCC**CCGGAATTGCCAGCTGGGGC
*kan*^*r*^-*Kpn*I_R	ATAT**GGTACC**TCAGAAGAACTCGTCAAGAA
*baeR*-up-*Sal*I_F	TTAA**GTCGAC**CGCCCGATTATGGTCAATAG
*baeR*-up-*Bam*HI_R	TATA**GGATCC**GCTTACTTCGAACCCAGCAG
*baeR*-dw-*Kpn*I_F	ATCG**GGTACC**TTGCTTAGAAAAGTTATGCT
*baeR*-dw-*Sac*I_R	AAAT**GAGCTC**ATGCTTTAGGGGTGGCTTCT
*baeR*-up-check_R	CTTTCCCAGTGGTGGTTACG
*baeR*-dw-check_R	GACGGACGTGGCTTACTCAT
pWH1266 check_F	TGCCACCTGACGTCTAAGAA
pWH1266 check_R	TCATACACGGTGCCTGACTG
*sacB*_F	AGTTTTGTTCAGCGGCTTGT
*sacB*_R	GGTCAGGTTCAGCCACATTT
*baeR*_F	GGATGGTTTAACGATTTGCC
*baeR*_R	TCCACTCATGACGGTTACCA
*baeS*_F	CTTTCCCAGTGGTGGTTACG
*baeS*_R	GAGCCAAGTCTGCCAAATCT
*baeS* probe_F	CCTCAATACTGGTGAAACCA
*baeS* probe_R	TCCCCCAATCATGATAAACG
*rpoB*_F	AGTCACGCGAAGTTGAAGGT
*rpoB*_R	GCGGTATGGAGTTTCCAAGA
16 s rRNA_F	GTAGCTTGCTACTGGACCTAG
16 s rRNA_R	CATACTCTAGCTCACCAGTATCG
*Kan*-2^*r*^-*EcoR*I_F	AATA**GAATTC**ACATCTCAACCATCATCG
*Kan*-2^*r*^-*EcoR*I_R	AATA**GAATTC**CATCTCAACCCTGAAGC
*baeR*-*Xho*I_F	TTAA**CTCGAG**CATGTTTCATGATGGTC
*baeR*-*Xba*I_R	TAGC**TCTAGA**TTATTCTTCTGGATATTCG
q*baeS*_F	GCCATTCAGCAGCATTCTTTC
q*baeS*_R	ATTTACCGTTCCCGCATCTG
q*baeR*_F	TGACAGCACGTACCGAAGAAA
q*baeR*_R	CATAATCATCTGCCCCCATGT
q*adeB*_F	ACAAGACCGCGCTAACTTAGGT
q*adeB*_R	TGCCATTGCCATAAGTTCATCT
q*adeA1*_F	CCTCAAGCGCTATTGGTTCCT
q*adeA1*_R	CCTGAGGCTCGCCACTGA
q*adeA2*_F	TTTGAGGCCGATGTAAATAGCA
q*adeA2*_R	GTCTTGCCACCTCAGCTTCAG
q16s rRNA_F	AGCATTTCGGATGGGAACTTTA
q16s rRNA_R	GTCGTCCCCGCCTTCCT

^a^Restriction sites introduced into the primers are highlighted using bold fonts and described in the primer name.

### Transcription of *baeR* and *baeS* under normal and stressed conditions

TCSs are commonly involved in stress responses in bacteria. Because no previous studies have explored the function of A1S_2883 and A1S_2884, we began by testing the response of both genes to high osmotic conditions to determine if they have functions that are similar to those of their BaeSR counterparts in other bacteria. To determine whether *A. baumannii baeSR* participates in the stress response, the relative levels of *baeR* and *baeS* transcription were detected in cells grown in Luria-Bertani (LB) agar (37°C, 220 rpm) with or without 20% sucrose. RT-PCR analysis showed that the expression levels of *baeR* and *baeS* were 2.3- and 6.7-fold higher in cells exposed to osmotic stress compared with cells grown without sucrose (Figure 2B). This result suggested that the BaeSR TCS in *A. baumannii* was involved in cellular adaptation to stress conditions such as high osmolarity.

### Construction of *baeR* deletion mutants and baeR-reconstituted strains

To further study the role of the BaeSR TCS in *A. baumannii*, in-frame deletion mutants of *baeR* were generated using the method of Sugawara et al. [23]. The successful construction of *baeR* deletion mutants was verified by PCR (Additional file 1: Figure S1B), RT-PCR (Additional file 2: Figure S2), and Southern blot assays (Additional file 3: Figure S3B). To generate the *baeR*-reconstituted strain, pWH1266-*kan*^*r*^-*baeR* was introduced into the *baeR* deletion mutant (AB1026; Table 2) by electroporation. The *baeR*-reconstituted strain was designated AB1027. In addition, pWH1266-*kan*^*r*^-*baeR* was also introduced into the wild-type strain to generate the strain AB1028. Successful construction of the AB1027 and AB1028 strains was verified by RT-PCR. The expression of *baeR* was comparable in the wild-type and the *baeR*-reconstituted AB1027 strains, whereas *baeR* was overexpressed in AB1028 relative to the wild-type strain (data not shown).

**Table 2:** Bacterial strains and plasmids used in this study

**Strain or plasmid**		**Relevant feature(s)**	**Source or reference**
*A. baumannii* strains	ATCC 17978	Wild-type strain	ATCC
	AB1026 (Δ*baeR*::*kan*^*r*^)	Derived from ATCC 17978. *baeR* mutant obtained by *kan*^*r*^ gene replacement	This study
	AB1027	AB1026 *baeR*::pWH1266	This study
	AB1028	ATCC 17978 *baeR*::pWH1266	This study
	AB1029	ATCC 17978 *kan*:: pWH1266	This study
	ABtc	Induced tigecycline resistant ATCC 17978	This study
	ABtcm (Δ*baeR*::*kan*^*r*^)	Derived from ABtc. *baeR* mutant obtained by *kan*^*r*^ gene replacement	This study
	ABhl1	Tigecycline resistant clinical isolate	This study
*E. coli* strains	XL1 blue	*recA1 endA1 gyrA96 thi-1 hsdR17 supE44 relA1 lac* [F’ *proAB lacI*^q^*Z*Δ*M15* Tn*10* (Tet^r^)]	Stratagene
	S17-1 (ATCC 47055)	*thi pro hsdR hsdM recA*[*RP42-Tc::Mu-* Km*::Tn7* (Tp^r^Sm^r^)Tra^+^]	ATCC
Plasmids	pEX18Tc	Suicide vector containing *sacB*, Tc^r^	40
	pSFS2A	Containing *kan*^*r*^, an FRT site, *FLP1*, and *CaSAT1* as a *SAT1* flipper	41
	pEX18Tc-Δ*bae*::*kan*^*r*^	pEX18Tc containing *baeR* upstream and downstream fragments joined by a *kan*^*r*^ cassette	This study
	pWH1266 (ATCC 77092)	*E. coli-A. baumannii* shuttle cloning vector, containing Amp^r^, Tet^r^	43
	pC2HP	Provided *kan*^*r*^ for pWH1266	42
	pWH1266-*kan*^*r*^	pWH1266 containing *kan*^*r*^	This study
	pWH1266-*kan*^*r*^-*baeR*	pWH1266-*kan*^*r*^ containing *baeR*	This study

### Minimal inhibitory concentration (MIC) determination

To correlate BaeR with tigecycline susceptibility, the MIC of tigecycline was determined. For *A. baumannii* ATCC 17978, the MIC of tigecycline was 0.5 μg/mL. However, the MIC of tigecycline for the *baeR* deletion mutant was 0.25 μg/mL; *baeR* reconstitution restored the MIC to the wild-type level (MIC 0.5 μg/mL). Moreover, the overexpression of *baeR* in AB1028 raised the MIC of tigecycline to 1 μg/mL. The introduction of pWH1266 alone did not affect the MIC of tigecycline, whereas the MICs obtained with the induced tigecycline-resistant strain ABtc and the clinical tigecycline-resistant strain ABhl1 were 256 and 16 μg/mL, respectively. These results indicate that BaeR is closely related to the tigecycline susceptibility of *A. baumannii*.

### Expression of the *adeAB* and *baeSR* genes in strains with different levels of tigecycline resistance

**Figure 3:**
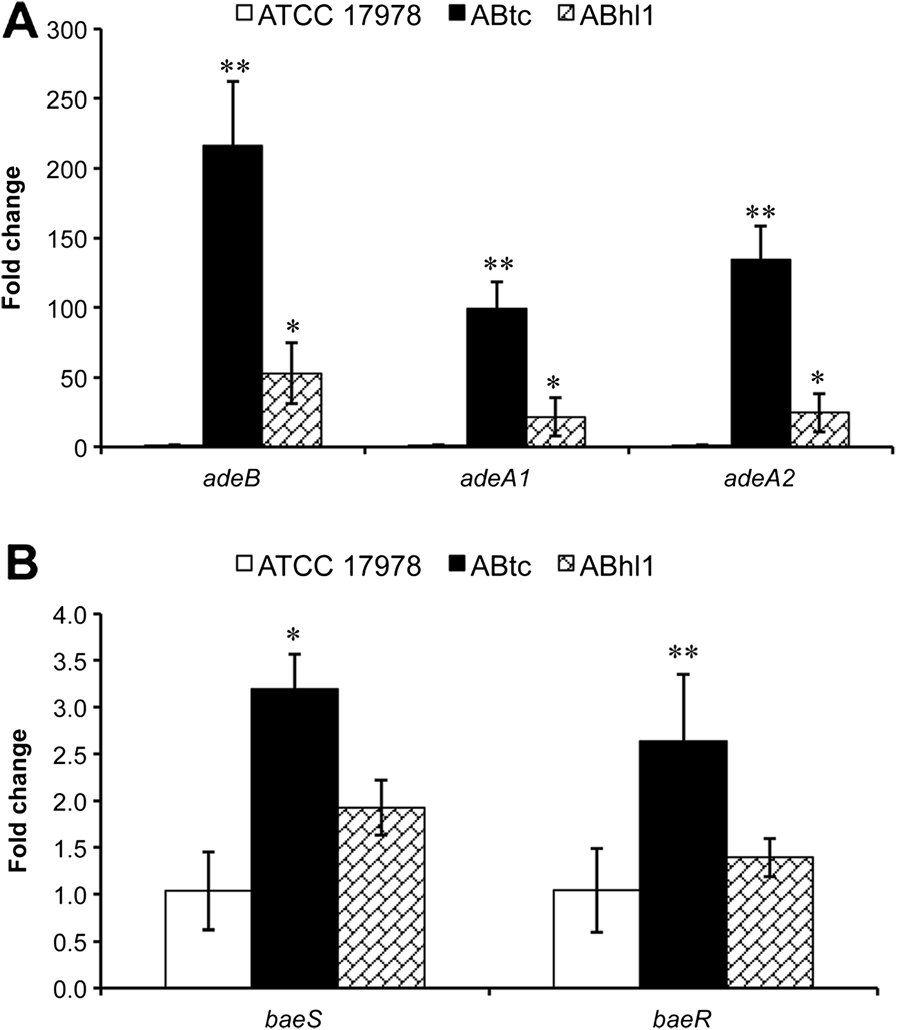
**Transcript levels of the *adeA*, *adeB*, *baeR*, and *baeS *genes in *A. baumannii*strains.** ABtc and ABhl1 are laboratory-induced and clinically isolated tigecycline-resistant strains, respectively. The corresponding tigecycline minimum inhibitory concentrations (MICs) of ATCC 17978, ABtc, and ABhl1 were 0.5, 256, and 16 μg/mL, respectively. Gene expression was detected by quantitative real-time PCR (qRT-PCR). **(A)** qRT-PCR showed that the expression levels of *adeB* in ABtc and ABhl1 were 216- and 53-fold higher than those in the wild-type strain, respectively. The *adeA1* expression levels in ABtc and ABhl1 were 99- and 22-fold higher than those in the wild-type strain, respectively, whereas the *adeA2* expression levels in ABtc and ABhl1 were 134- and 25-fold higher. **(B)** The expression levels of *baeS* and *baeR* in ABtc increased 3.19 and 2.64 times, respectively, compared with the wild-type strain, whereas those in ABhl1 only increased 1.93 and 1.39 times, respectively. *16S rRNA* gene was used as a control. The results are displayed as the means ± SD from four independent experiments. *, *P* < 0.05; **, *P* < 0.01.

To further decipher the role of the BaeSR TCS and AdeAB in tigecycline resistance, we analyzed gene expression in the wild-type *A. baumannii* strain ATCC 17978 as well as the ABtc and ABhl1 strains. The quantitative real-time PCR (qRT-PCR) results showed that the expression levels of *adeB* were 216- and 53-fold higher in ABtc and ABhl1, respectively, than in the wild-type strain. ABtc and ABhl1 also showed increased transcription of *adeA1* and *adeA2*, although it was less marked than that of *adeB* (Figure 3A). The expression levels of *baeS* and *baeR* in ABtc increased 3.19 and 2.64-fold, respectively, compared with the wild-type strain, whereas those in ABhl1 only increased 1.93 and 1.39-fold, respectively (Figure 3B). Overall, the combination of the qRT-PCR results with the MIC assay above suggest that both BaeSR and AdeAB are involved in the tigecycline resistance of *A. baumannii*.

### Influence of the BaeSR TCS on *adeAB* efflux pump expression

**Figure 4:**
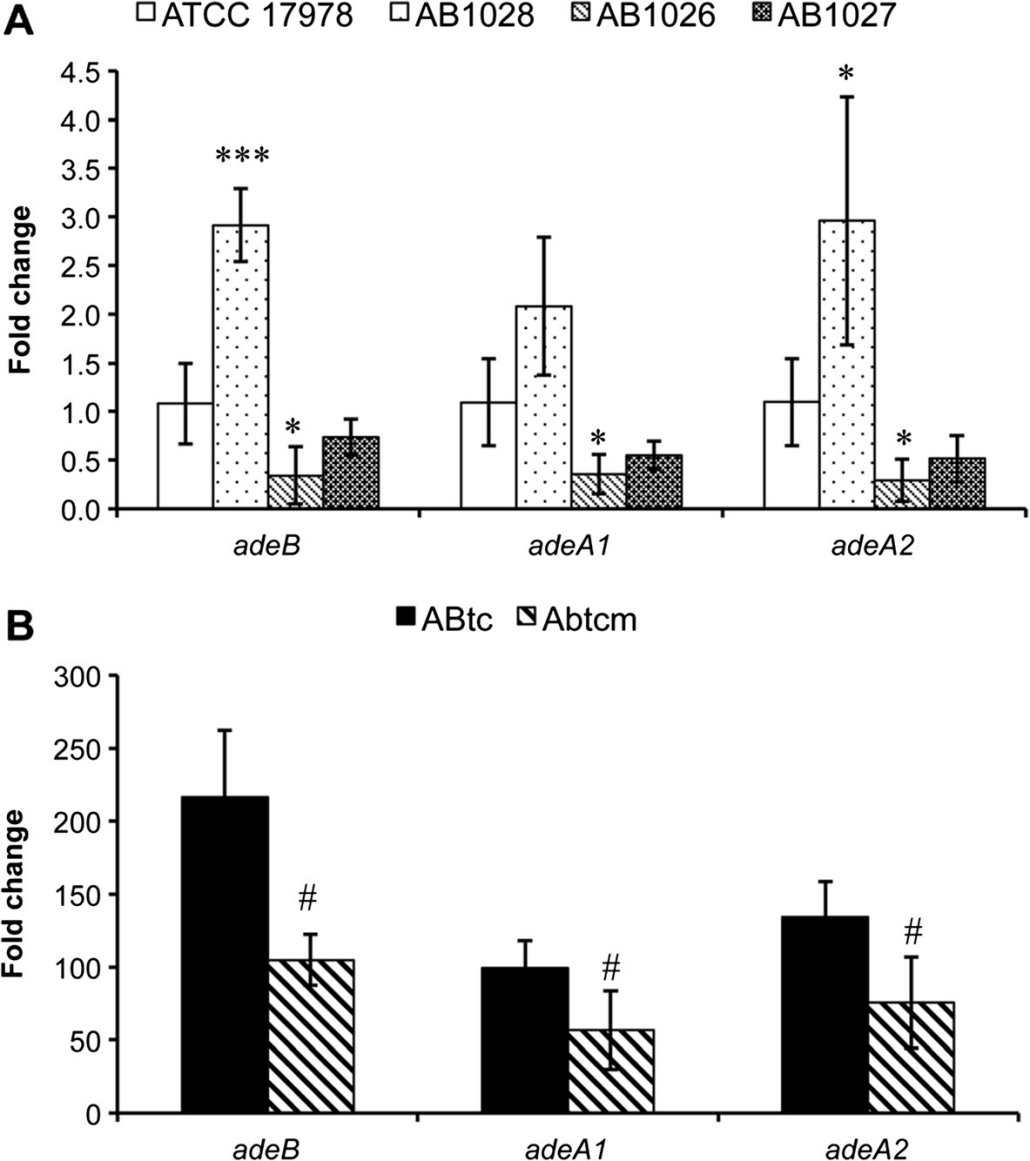
**Transcript levels of the *adeA *and *adeB *genes in different strains of *A. baumannii*.** AB1026, AB1027, and AB1028 are the *baeR* deletion mutant, *baeR* reconstitution, and wild-type with *baeR* overexpression strains, respectively. ABTcm is the *baeR* deletion mutant of ABtc, which was a laboratory-induced tigecycline-resistant strain. The relative expression of *adeB*, *adeA1*, and *adeA2* was determined by qRT-PCR. **(A)** The expression levels of *adeB*, *adeA1*, and *adeA2* in AB1028 were approximately 2.9-, 2.1-, and 3-fold higher, respectively, than those in ATCC 17978, while the deletion of *baeR* in the wild-type strain decreased the expression levels of these three pump genes by 68.3%, 67.3%, and 73.5%, respectively. The decreased expression of the pump genes can be partially restored by *baeR* reconstitution. **(B)** The expression levels of *adeB*, *adeA1*, and *adeA2* in ABtcm were 51.5%, 42.7%, and 43.7% lower, respectively, than those in ABtc. *16S rRNA* gene was used as a control. The results are displayed as the means ± SD from three independent experiments. *, *P* < 0.05; ***, *P* < 0.001. #, *P* < 0.05 between ABtc and ABtcm.

To understand whether *baeR* influenced the tigecycline MIC by affecting the *adeAB* efflux pump gene, the expression of *adeA1*, *adeA2*, and *adeB* in ATCC 17978, AB1026, AB1027, and AB1028 was analyzed by qRT-PCR*.* The expression levels of *adeB*, *adeA1*, and *adeA2* in AB1028 were approximately 2.9-, 2.1-, and 3-fold higher, respectively, than those in ATCC 17978, while the deletion of *baeR* from the wild-type strain decreased the expression levels of these three pump genes by 68.3%, 67.3%, and 73.5%, respectively (Figure 4A). The decreased expression of the pump genes can be partially restored by *baeR* reconstitution (Figure 4A). To determine the impact of *baeR* deletion on *adeR* expression, RT-PCR was also performed. No differences in *adeR* expression were observed between AB1026 and the wild-type strain (data not shown). Overall, these findings suggest that BaeR upregulates the expression of *adeAB* genes.

### Expression analysis of *adeAB* in induced tigecycline-resistant A. baumannii and its *baeR* mutant

To further confirm the role of *baeR* in the tigecycline resistance of *A. baumannii* via the AdeAB efflux pump, a *baeR* deletion mutant of ABtc (ABtcm) was constructed and *adeAB* expression was analyzed by qRT-PCR. The expression levels of *adeB*, *adeA1*, and *adeA2* in ABtcm were 51.5, 42.7%, and 43.7% lower, respectively, than those in ABtc (Figure 4B). These data confirmed the contribution of BaeR to the regulation of AdeAB, which is essential to tigecycline resistance in *A. baumannii*.

### Time-kill assay

**Figure 5:**
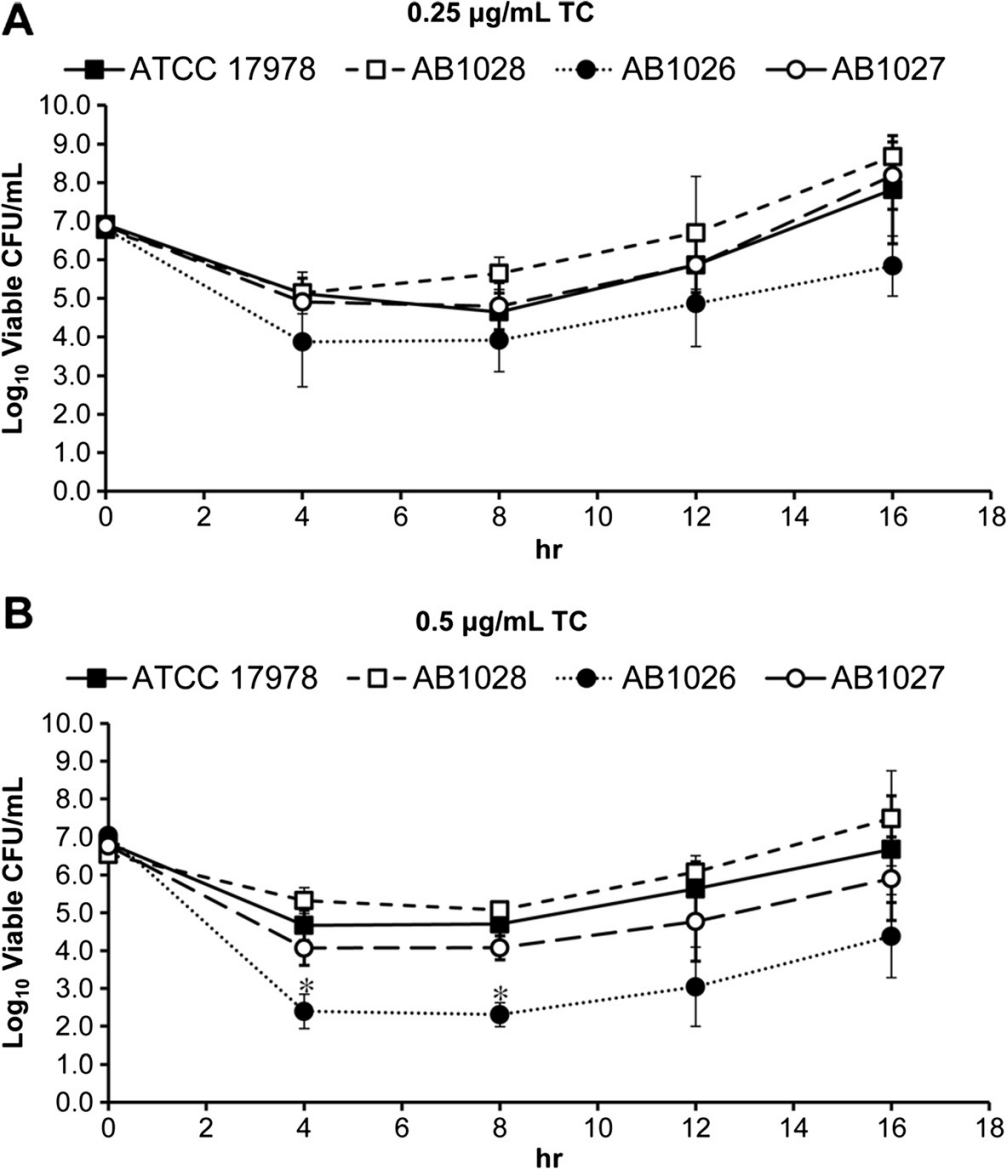
**Time-kill assays for ATCC 17978, AB1026, AB1027, and AB1028 with 0.25 μg/mL (A) and 0.5 μg/mL (B) tigecycline.** In the presence of 0.25 μg/mL tigecycline, all tested strains showed similar surviving colony forming unit (CFU) curves, in which the lowest value occurred at 4 h and was followed by regrowth. AB1026 had a greater CFU reduction than the wild-type strain throughout the assay period, which could be restored by *baeR* reconstitution. Increasing the tigecycline concentration to 0.5 μg/mL resulted in a marked 4.7-log_10_ CFU reduction for AB1026 at 8 h, which was followed by regrowth, whereas a smaller reduction was observed for the wild-type strain. *baeR* reconstitution did not fully restore the ability of AB1026 to resist 0.5 μg/mL tigecycline. AB1028 had a slight, though not significant, CFU reduction compared to the wild-type strain in the presence of 0.25 or 0.5 μg/mL tigecycline. Viable counts represented by CFUs were determined at time 0 and at 4, 8, 12, and 16 h after inoculation. A time-kill curve was constructed for each strain. The results are displayed as the means ± SD from three independent experiments. *, *P* < 0.05.

To further compare the effects of BaeR on tigecycline susceptibility, time-kill assays were performed using ATCC 17978, AB1026, AB1027, and AB1028. There were no differences in the surviving colony forming units (CFUs) among these four strains when tigecycline was not added to the LB agar. In the presence of 0.25 μg/mL tigecycline, all tested strains had similar surviving CFU curves; the lowest value was observed at 4 h, which was followed by regrowth (Figure 5A). Additionally, AB1026 showed a greater reduction in CFUs than the wild-type strain (e.g., 2.9-log_10_ versus 1.8-log_10_ reduction, respectively, at 4 h) throughout the assay period, which could be restored by *baeR* reconstitution. Increasing the tigecycline concentration to 0.5 μg/mL produced an even more marked 4.7-log_10_ reduction in the CFUs of AB1026 at 8 h, which was followed by regrowth. In contrast, a smaller reduction (2.1-log_10_ reduction at 8 h) was observed for the wild-type strain (Figure 5B). However, *baeR* reconstitution did not fully restore the ability of AB1026 to resist 0.5 μg/mL tigecycline. AB1028 showed a slightly smaller reduction in CFUs than the wild-type strain in the presence of 0.25 and 0.5 μg/mL tigecycline. Therefore, the time-kill assay indicates that the BaeSR TCS plays a role in the tigecycline susceptibility of *A. baumannii*.

## Discussion

Previous studies that investigated the regulation of AdeABC efflux pumps in *A. baumannii* primarily focused on the AdeRS TCS, which is located upstream of the *adeABC* operon and is transcribed in the opposite direction [15]. Several point mutations in *adeR* or *adeS* have been proposed as the major cause of AdeABC efflux pump overexpression, including a threonine-to-methionine substitution at position 153 [15], a glycine-to-aspartate mutation at position 30 [24], an alanine-to-valine substitution at position 94 of AdeS [25], or a proline-to-leucine substitution at position 116 of AdeR [15]. However, the effect of AdeR or AdeS mutations on the expression of AdeABC is not always consistent. Different tigecycline MICs were observed in two transformed strains with the same mutations in the DNA-binding domain of the AdeR protein [16]. *adeABC*-overexpressing mutants that did not carry any mutations in *adeRS* compared with their isogenic parents were also reported [7,25]. Another mechanism leading to the overexpression of AdeABC involves the transposition of an IS*Aba1* copy into *adeS*[15], which stimulates AdeR to interact with and activate the *adeABC* promoter [16]. In contrast to the results of the above-mentioned studies of AdeRS, four imipenem-resistant *A. baumannii* strains carrying *adeB* but lacking *adeRS* were identified by Hou et al. [26], suggesting that another regulatory mechanism may be involved. Henry et al. reported that BaeSR was associated with the increased expression of the multidrug resistance-associated efflux pump genes *macAB-tolC* and *adeIJK* in their transcriptional analysis of lipopolysaccharide-deficient *A. baumannii* 19606R [27]. Therefore, the role of BaeSR in the expression of the AdeABC efflux pump deserves investigation. Our data demonstrate that BaeSR influences the tigecycline susceptibility of *A. baumannii* ATCC 17978 through its positive regulation of the transcription of transporter genes *adeA* and *adeB*. This result supported the possibility that other TCSs aside from AdeRS may be involved in the regulation of the AdeABC efflux pump in *A. baumannii*.

Most *A. baumannii* strains have an RND efflux pump, AdeABC, which has a three-component structure with AdeB forming the transmembrane component, AdeA forming the inner membrane fusion protein, and AdeC forming the outer membrane protein [9]. However, according to the NCBI GenBank database, *A. baumannii* ATCC 17978 lacks an *adeC* gene but has two *adeA* genes and one *adeB* gene. *A. baumannii* AYE, *A. baumannii* ACICU, *A. baumannii* ATCC 19606, and *A. baumannii* TYTH-1 all possess an AdeC-like outer membrane protein. Marchand et al. constructed a clinical *A. baumannii* strain with an inactivated *adeC*. This derivative mutant displayed resistance to the various substrates of the AdeABC pump that was similar to that of the wild-type strain, indicating that *adeC* is not essential for resistance [15]. Because *adeC* was not found in 41% of the clinical isolates carrying *adeRS-adeAB* in one study [28], it is reasonable to deduce that AdeAB could recruit another outer membrane protein to form a functional tripartite complex [29].

The first description of tigecycline non-susceptibility was reported by Peleg et al. [7]. These authors found that the efflux pump inhibitor phenyl-arginine-β-naphthylamide could cause a four-fold reduction in the MIC of tigecycline in two tigecycline-non-susceptible isolates. The qRT-PCR results showed 40-fold and 54-fold increases in *adeB* expression in these two isolates compared to that observed in a tigecycline-susceptible isolate. Their finding is consistent with our comparison of tigecycline MICs and expression levels of AdeAB among the wild-type, ABhl1, and ABtc strains. Despite the important role of AdeABC in antibiotic resistance, this efflux pump operon is cryptic in natural isolates of *A. baumannii*[15,30]. Antibiotic exposure, including exposure to tigecycline, could induce pump overexpression, resulting in drug resistance [29]; this was observed in our ABtc strain. Furthermore, there was a statistically significant linear relationship between log-transformed *adeA* expression values and log-transformed MICs of tigecycline in clinical isolates of the *A. calcoaceticus-A. baumannii* complex, indicating that the overexpression of the AdeABC efflux pump is a prevalent mechanism for this resistance phenotype [31].

The modest increase in AdeAB pump gene expression in AB1028 relative to the wild-type strain may have been due to the overexpression of BaeSR. However, because ABtcm had only moderately reduced *adeB*, *adeA1*, and *adeA2* expression levels relative to ABtc, we proposed that control mechanisms aside from BaeSR, such as sequence changes in *adeR* or *adeS*, were responsible for the overexpression of these pump genes. The regulators that are involved in efflux gene expression are either local or global regulators [32]. One of the most well-studied examples is the AcrAB-TolC system of *E. coli*[33]. This system is under the control of the local repressor gene *acrR*, which negatively regulates the transcription of *acrAB*. On the other hand, global stress conditions are assumed to result in the generation of global transcription regulators. These regulators are unlikely to be MarA, SoxS, or Rob, but could be their homologs. Such regulators increase the transcription of not only *acrAB* but also *acrR*, which functions as a secondary modulator to repress *acrAB*. Fernando et al. demonstrated that the transcription patterns of both *adeB* and *adeJ* are cell density-dependent and similar, indicating a role for global regulatory mechanisms in the expression of these genes in *A. baumannii*[34]. Two-component regulatory systems mediate the adaptive responses of bacterial cells to a broad range of environmental stimuli [35]. In this study, qRT-PCR analysis of *baeSR* expression under high sucrose conditions suggested that this TCS was involved in the regulation related to this stress condition. Therefore, we propose that BaeSR, which functions as an envelope stress response system to external stimuli, also influences the transcription of *adeAB* in *A. baumannii* by functioning as a regulator of global transcription. Meanwhile, the well-described *adeR* is an example of a local regulator that activates *adeABC* expression [15,16]. However, the relationship between BaeSR and AdeRS must be further clarified. Because the expression of *adeRS* was only marginally increased in the *baeSR* deletion mutants in this study, we assume that the crosstalk between these TCSs might be absent or only very weak. The question of whether other TCSs are involved in the regulation of the AdeABC efflux pump and how they interact in *A. baumannii* merits further investigation.

## Conclusions

In this study, we showed for the first time that the BaeSR TCS influences the tigecycline susceptibility of *A. baumannii* by positively regulating the RND efflux pump genes *adeA* and *adeB*. However, whether BaeSR can also contribute to tigecycline resistance through other transporter genes, such as *macAB-tolC* and *adeIJK*, is not yet clear, and related studies are underway. Overall, this finding highlights the complexity of AdeABC transporter regulation and could be a starting point for understanding the role of TCSs in the antimicrobial susceptibility of bacteria.

## Methods

### Bacterial strains, plasmids, growth conditions, and antibiotic susceptibility testing

The bacterial strains and plasmids used in this study are listed in Table 2. The cells were grown at 37°C in LB broth and agar. To determine the MIC, a broth microdilution method was used according to the 2012 CLSI guidelines [36]. Briefly, bacteria were inoculated into 1 mL cation-adjusted Mueller-Hinton broth (CAMHB) (Sigma-Aldrich, St. Louis, MO) containing different concentrations of tigecycline (Pfizer, Collegeville, PA) to reach ≈ 5 × 10^5^ CFU/mL, and the cultures were incubated at 37°C for 24 h. The lowest tigecycline concentration that completely inhibited bacterial growth was defined as the MIC, and growth was determined by unaided eyes and by measuring optical densities (ODs) using a spectrophotometer. On the basis of the report published by Pachón-Ibáñez et al., the provisional MIC breakpoints for tigecycline are ≤2, 4, and ≥8 μg/mL to designate susceptible, intermediate, and resistant strains, respectively [37].

### DNA manipulation

Plasmid DNA was prepared with the FavorPrep™ Plasmid DNA Extraction Mini Kit (Favorgen, Ping-Tung, Taiwan). *A. baumannii* genomic DNA was extracted as described previously [38]. PCR amplification of the DNA was performed in a Thermo Hybaid PXE 0.2 HBPX02 Thermal Cycler (Thermo Scientific, Redwood, CA), using Pro*Taq™* DNA Polymerase (Protech, Taipei, Taiwan) or the KAPA HiFi™ PCR Kit (Kapa Biosystems, Boston, MA). DNA fragments were extracted from agarose gels and purified using the GeneKlean Gel Recovery & PCR CleanUp Kit (MDBio, Inc., Taipei, Taiwan). Nucleotide sequences of the PCR products were verified using an ABI 3730XL DNA Analyzer (Applied Biosystems, South San Francisco, CA).

### RNA isolation, RT-PCR, and qRT-PCR

For total RNA isolation, *A. baumannii* ATCC 17978 was grown overnight in LB broth (37°C, 220 rpm, 16 h) to reach an OD_600_ of approximately 6.5. The overnight cultures were sub-cultured at a 1:100 dilution in 25 mL fresh LB medium. The cells were grown to mid-log phase and harvested by centrifugation at 4°C. The cell pellets were resuspended in 200 μL ice-cold RNA extraction buffer (0.1 M Tris-Cl [pH 7.5], 0.1 M LiCl, 0.01 M ethylenediaminetetraacetic acid [pH 8.0], 5% sodium dodecyl sulfate [SDS], 2% β-mercaptoethanol), and 200 μL ice-cold phenol-chloroform-isoamyl alcohol (PCIA [25:24:1], pH 4.5) was added and vortexed for 2 min. The supernatants were then collected by centrifugation, added to 200 μL ice-cold PCIA, and mixed well. This step was repeated three times. Then, RNA was precipitated with ethanol at -80°C overnight and collected by centrifugation at maximum speed for 5 min. The RNA pellets were dissolved in 25–100 μL diethylpyrocarbonate-treated water. DNA was removed using Ambion® TURBO™ DNase (Life Technologies, Grand Island, NY), and cDNA was synthesized by reverse transcription using High-Capacity cDNA Reverse Transcriptase Kits (Applied Biosystems). The cDNAs were used in PCR reactions with different primers (Table 1).

qRT-PCR was carried out with a StepOne™ Real-Time PCR System (Life Technologies). The primers used for qRT-PCR are listed in Table 1. Briefly, each 20-μL reaction mixture contained 25 ng cDNA, 10 μL Power SYBR green PCR master mix (Life Technologies), and 300 nM each forward and reverse primer. The reactions were performed with 1 cycle at 95°C for 10 min followed by 40 cycles of 95°C for 15 s and 60°C for 1 min. The 16S rRNA transcript was used as an endogenous control for the qRT-PCR. The data were analyzed using StepOne v2.1 software (Life Technologies).

### Induction of tigecycline resistance

To induce tigecycline resistance, serial passaging was performed as previously described [39] with some modifications. Briefly, on day 1, 3 mL of LB broth containing tigecycline at the MIC was inoculated with *A. baumannii* (passage 1), and the cultures were incubated at 37°C with shaking (220 rpm). On day 3, 30 μL of the culture was transferred to 3 mL of LB broth containing tigecycline at 8× the MIC (passage 2), and the cultures were again incubated at 37°C with shaking (220 rpm). On day 5, 30 μL of the culture was transferred into LB broth containing tigecycline at 16× the MIC (passage 3), and the cultures were again incubated at 37°C with shaking (220 rpm). This passaging was repeated on day 7 (passage 4). On day 9, aliquots (3 mL) of the cultures were mixed with 10% glycerol and stored at -80°C until use. Daily passaging in tigecycline-free LB was conducted for 30 days for both ATCC 17978 and the clinical strain.

### Construction of *baeR* deletion mutants and baeR reconstituted strains

To assess the contribution of BaeR to the regulation of tigecycline resistance, *baeR* deletion mutants of *A. baumannii* ATCC 17978 were constructed as previously described [23] with some modifications. The suicide vector pEX18Tc [40] was first cloned with a 953-bp DNA fragment carrying a kanamycin resistance cassette, which was PCR-amplified from the pSFS2A plasmid [41], to generate pEX18Tc-*kan*^*r*^. DNA fragments carrying the upstream and downstream regions of the *baeR* gene, referred to as *baeR*-up and *baeR*-dw, were independently amplified by PCR using the primer pairs *baeR*-up-*Sal*I-F and *baeR*-up-*Bam*HI-R or *baeR*-dw-*Kpn*I-F and *baeR*-dw-*Sac*I-R (Table 1). The *baeR*-up fragment (1,119 bp) was digested with *Sal*I and *Bam*HI enzymes, whereas the *baeR*-dw fragment (1,120 bp) was digested with *Kpn*I and *Sac*I enzymes (Additional file 4: Figure S4A). Both enzyme-digested DNA fragments were then independently cloned into the corresponding restriction sites of pEX18Tc-*kan*^*r*^, generating pEX18Tc-*kan*^*r*^-*baeR-*flanking. The resultant plasmid was then transformed into the *E. coli* S17-1 strain using the standard CaCl_2_/heat shock method [38]. Then, trans-conjugation was performed between *E. coli* S17-1 donor cells and *A. baumannii* ATCC 17978 recipient cells to transfer and integrate pEX18Tc-*kan*^*r*^-*baeR*-flanking into the chromosome of ATCC 17978 (Additional file 4: Figure S4B). By growing the ATCC 17978 conjugate cells on LB agar containing 10% sucrose, the cells were able to resolve the suicide plasmid pEX18Tc (Additional file 4: Figure S4C). Sucrose-resistant colonies were examined to verify that they had the kanamycin-resistant phenotype as a result of plasmid eviction. The absence of the *baeR* gene sequence in the genome was verified by PCR and RT-PCR and further confirmed by Southern blot hybridization.

To reconstitute the *baeR* gene in the *baeR*-deleted mutants, a DNA fragment carrying the entire *baeR* gene sequence was generated by PCR using the genomic DNA of *A. baumannii* ATCC 17978 as the template. Briefly, a kanamycin resistance cassette was first amplified from the pC2HP vector [42] and cloned into the *E. coli*/*Acinetobacter* shuttle vector pWH1266 [43,44] (Additional file 5: Figure S5A and S5B). Subsequently, the *baeR* DNA fragment was cloned into the *Xba*I/*Xho*I restriction sites (Additional file 5: Figure S5C). The plasmid was transformed into the wild-type strain and the *baeR* deletion mutants by electroporation, thus creating the *baeR*-overexpressing strain and complemented mutant strains, respectively. The overexpression and *baeR*-reconstituted strains were selected on LB agar containing 10 μg/mL tetracycline and were further verified by PCR (Additional file 5: Figure S5D) and RT-PCR (Additional file 2: Figure S2).

### Southern blot hybridization

Southern blot analysis was performed as reported in a previous publication [45]. Genomic DNA was extracted, and approximately 10 μg was digested with *Bcl*I overnight at 50°C. The DNA was then separated on a 0.8% agarose gel containing 1:10,000 SYBR Safe gel stain (Invitrogen, Grand Island, NY), transferred onto a positively charged nylon membrane (Pall Corporation, Port Washington, NY) via the alkaline transfer method [38], and fixed by baking at 80°C for 2 h. The membrane was hybridized with an [α-^32^P] dCTP-labeled *baeS* probe (Additional file 3: Figure S3A) using prehybridization buffer (6× saline sodium citrate [SSC; 1× SSC is 0.15 M NaCl plus 0.015 M sodium citrate], 5× Denhardt’s reagent, 0.5% SDS, 100 μg/mL salmon sperm DNA, and 50% formamide) at 42°C overnight. The membrane was then washed and visualized by autoradiography.

### Time-kill assay

The time-kill assays were carried out in duplicate as previously described [46] with some modifications. Briefly, cells were grown to log phase and sub-cultured into 10 mL CAMHB broth without (control) or with tigecycline (0.25 or 0.5 μg/mL) to a cell density of approximately 5 × 10^5^ CFU/mL. The cultures were incubated in an ambient atmosphere at 37°C. At different time points (0, 4, 8, 12, and 16 h) after inoculation, 0.1 mL of the culture was removed from each tube and 10-fold serially diluted. Then, 25 μL of each diluted cell suspension was spotted onto LB agar in duplicate. Viable cell counts were determined, the duplicates were averaged, and the data were plotted.

## Competing interests

The authors declare that they have no competing interests.

## Authors’ contributions

MFL conceived the study design and drafted the manuscript. YYL performed the laboratory work, including the mutant construction and complementation, gene expression, and time-kill assays. HWL carried out the MIC determinations. CYL participated in the overall design of this study and assisted in writing the manuscript. All authors have read and approved the final manuscript.

## Supplementary Material

Additional file 1: Figure S1. Verification of the *baeR* deletion mutants. **(A)** Diagram of the *baeR* gene and deletion mutant verification using appropriate primers. **(B)** Successful *baeR* gene fragment deletion was deduced based on a change in the PCR band size from 4539 bp to 4884 bp. Click here for file

Additional file 2: Figure S2. Southern blot analysis. **(A)** Genomic DNA from the *baeR* deletion mutant and the parental strain was digested by *Bcl*I. The location of the specific DNA probe is shown. **(B)** The bands corresponding to 6.7-kb and 2.8-kb fragments are indicated. Four independent clones of AB1026 are included. Click here for file

Additional file 3: Figure S3. Construction of the *baeR* deletion mutant. **(A)** A single crossover between pEX18Tc containing *baeR* upstream and downstream sequences joined by a *kan*^*r*^ cassette and the ATCC 17978 chromosome. **(B)** Two mechanisms by which the plasmid can integrate into the chromosome are diagrammed. **(C)** The suicide plasmid was excised by 10% sucrose counter-selection and selection of the in-frame *baeR* deletion strain with kanamycin. Click here for file

Additional file 4: Figure S4. Shuttle vector pWH1266 and verification of pWH1266 introduction into different strains of *Acinetobacter baumannii.***(A)** pWH1266. **(B)** pWH1266 with kanamycin cassette insertion. **(C)***baeR* insertion into the *Xba*I/*Xho*I restriction sites in pWH1266. **(D)** Successful *baeR* gene fragment insertion into the kanamycin cassette was deduced based on a change in the PCR band size from 1375 bp to 983 bp. AB1027, AB1028, and AB1029 represent the *baeR* reconstituted strain, the *baeR*-overexpressing strain, and the *A. baumannii* ATCC 17978 strain with pWH1266, respectively. Click here for file

Additional file 5: Figure S5. *baeR* gene expression in different *A. baumannii* strains as determined by reverse transcription polymerase chain reaction. No *baeR* expression could be observed in AB1026. AB1027 was the *baeR*-reconstituted strain derived from AB1026, which had a *baeR* expression level similar to that of the wild-type strain. AB1028 and AB1029 represent the *baeR*-overexpressing strain and *A. baumannii* ATCC 17978 with pWH1266, respectively. Click here for file
