# Supplementary figures and images for: Role of the BaeSR two-component system in the regulation of Acinetobacter baumannii adeAB genes and its correlation with tigecycline susceptibility
Source: BMC Microbiol. 2014 May 9;14:119. doi: 10.1186/1471-2180-14-119 (PMC4101873; doi:10.1186/1471-2180-14-119)

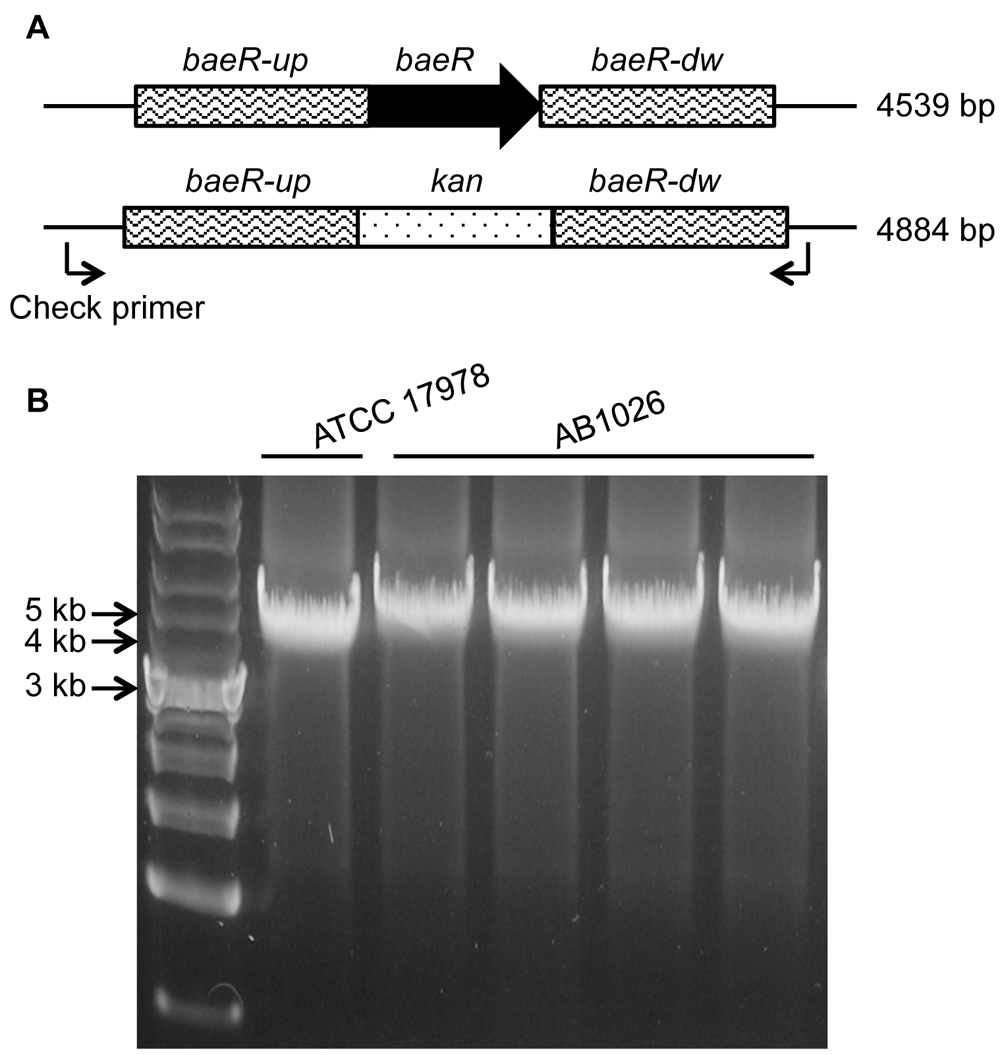

Supplement: Additional file 1: Figure S1. — Verification of the baeR deletion mutants. (A) Diagram of the baeR gene and deletion mutant verification using appropriate primers. (B) Successful baeR gene fragment deletion was deduced based on a change in the PCR band size from 4539 bp to 4884 bp. [file 1471-2180-14-119-S1.tiff]

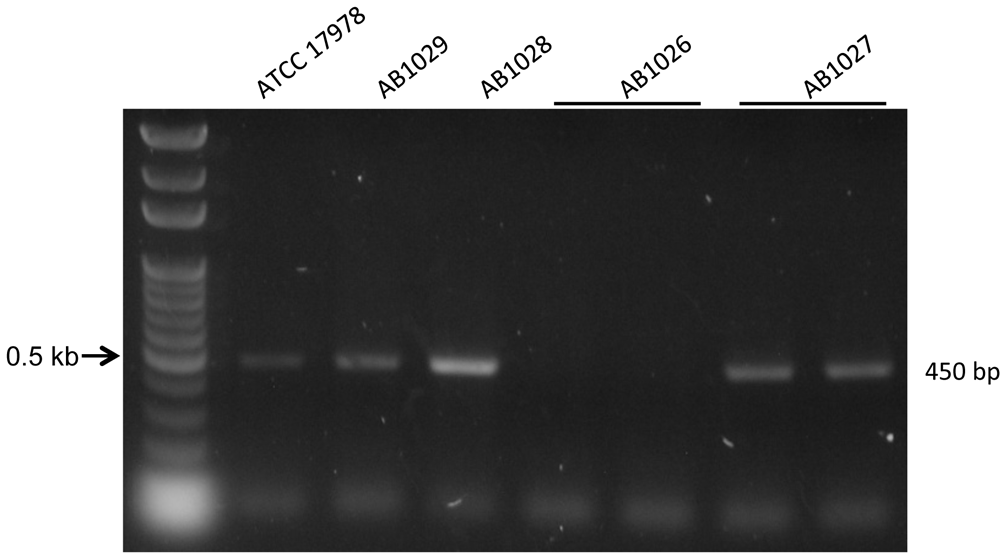

Supplement: Additional file 2: Figure S2. — Southern blot analysis. (A) Genomic DNA from the baeR deletion mutant and the parental strain was digested by BclI. The location of the specific DNA probe is shown. (B) The bands corresponding to 6.7-kb and 2.8-kb fragments are indicated. Four independent clones of AB1026 are included. [file 1471-2180-14-119-S2.tiff]

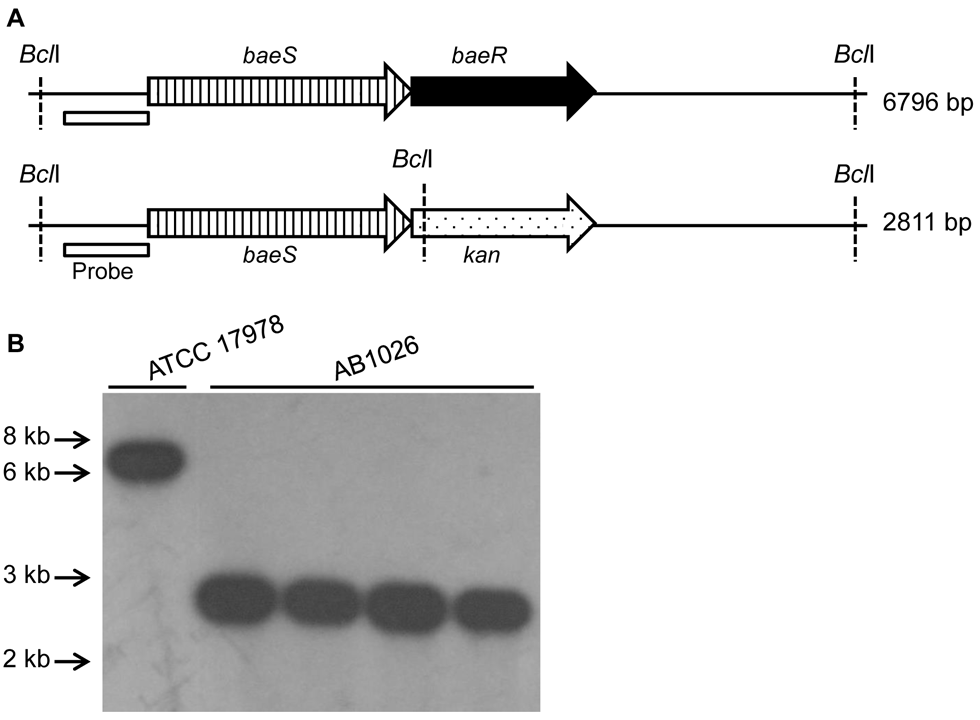

Supplement: Additional file 3: Figure S3. — Construction of the baeR deletion mutant. (A) A single crossover between pEX18Tc containing baeR upstream and downstream sequences joined by a kan r cassette and the ATCC 17978 chromosome. (B) Two mechanisms by which the plasmid can integrate into the chromosome are diagrammed. (C) The suicide plasmid was excised by 10% sucrose counter-selection and selection of the in-frame baeR deletion strain with kanamycin. [file 1471-2180-14-119-S3.tiff]

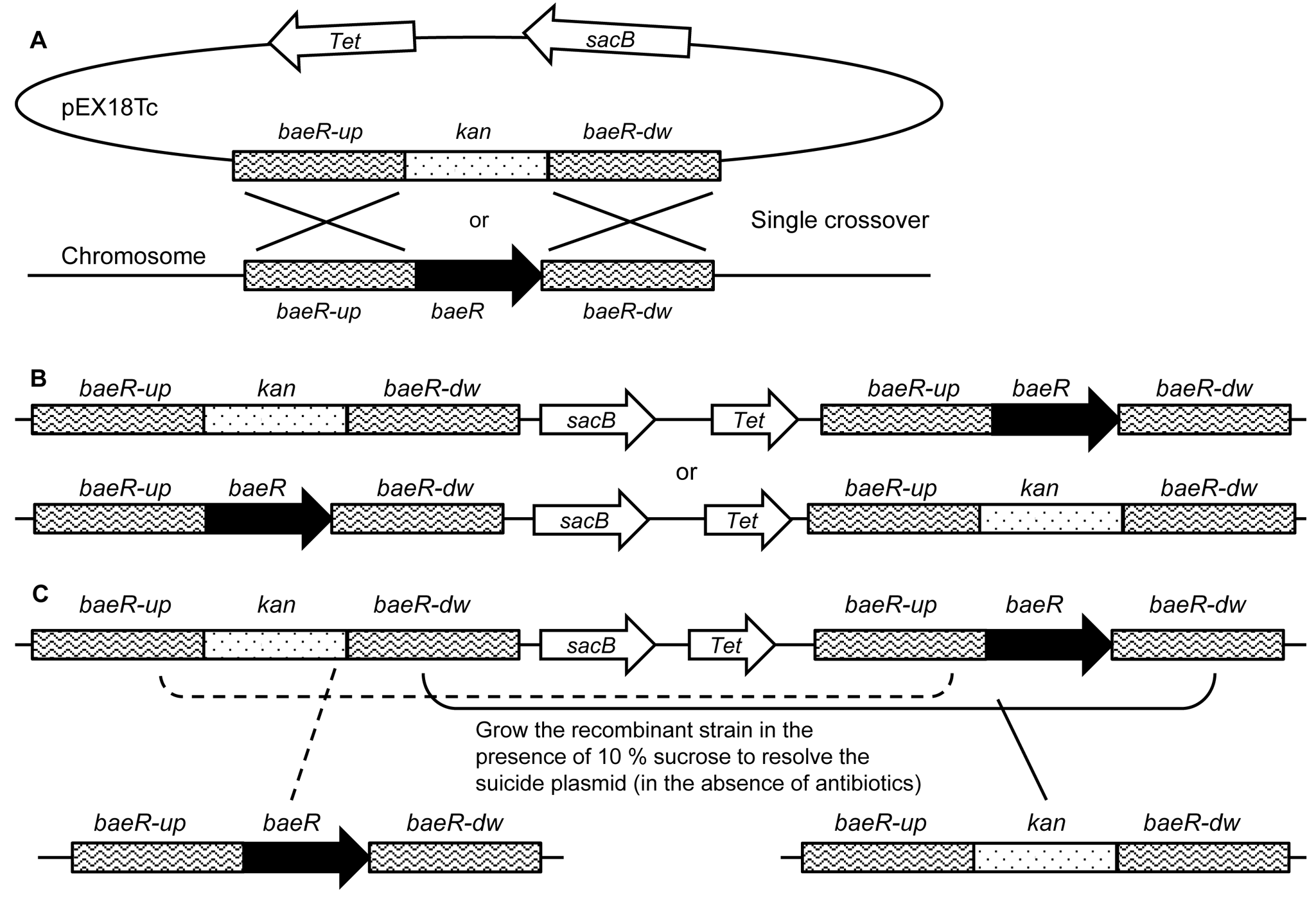

Supplement: Additional file 4: Figure S4. — Shuttle vector pWH1266 and verification of pWH1266 introduction into different strains of Acinetobacter baumannii.(A) pWH1266. (B) pWH1266 with kanamycin cassette insertion. (C)baeR insertion into the XbaI/XhoI restriction sites in pWH1266. (D) Successful baeR gene fragment insertion into the kanamycin cassette was deduced based on a change in the PCR band size from 1375 bp to 983 bp. AB1027, AB1028, and AB1029 represent the baeR reconstituted strain, the baeR-overexpressing strain, and the A. baumannii ATCC 17978 strain with pWH1266, respectively. [file 1471-2180-14-119-S4.tiff]

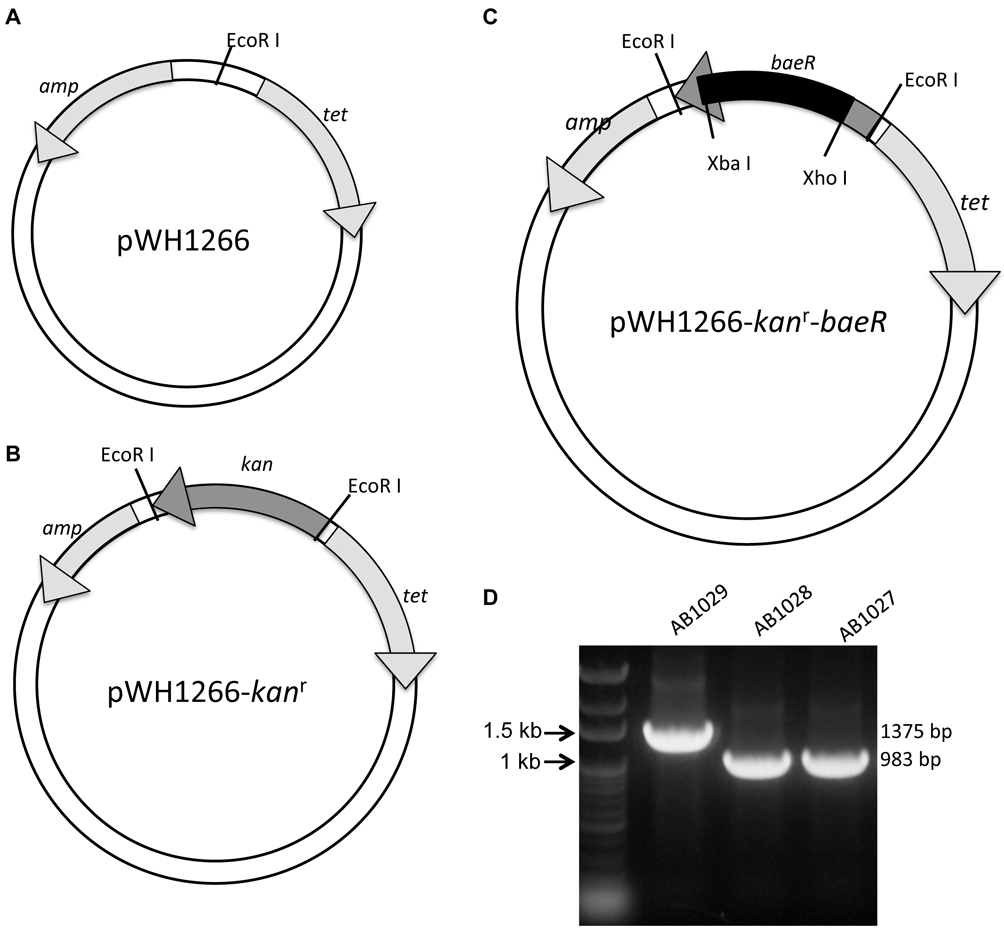

Supplement: Additional file 5: Figure S5. — baeR gene expression in different A. baumannii strains as determined by reverse transcription polymerase chain reaction. No baeR expression could be observed in AB1026. AB1027 was the baeR-reconstituted strain derived from AB1026, which had a baeR expression level similar to that of the wild-type strain. AB1028 and AB1029 represent the baeR-overexpressing strain and A. baumannii ATCC 17978 with pWH1266, respectively. [file 1471-2180-14-119-S5.tiff]
